# Seroepidemiology and Reactivation Rates of Cytomegalovirus in HIV-Positive Patients in Istanbul: A Retrospective Analysis

**DOI:** 10.3390/v18030394

**Published:** 2026-03-21

**Authors:** Derya Sevimli Saydan, Murat Hakan Kir, Muammer Osman Köksal, Kutay Sarsar, Arat Hulikyan, Atahan Cagatay, Mehmet Demirci, Pınar Soguksu, Eray Yurtseven, Serra Zeynep Akkoyunlu, Sevim Meşe, Ali Agacfidan, Hayriye Kirkoyun Uysal

**Affiliations:** 1 Department of Medical Microbiology, Istanbul Faculty of Medicine, Istanbul University, 34093 Istanbul, Turkey; deryasevimli1@gmail.com (D.S.S.); muammerosmankoksal@istanbul.edu.tr (M.O.K.); kutay.sarsar@istanbul.edu.tr (K.S.); arathulikyan@hotmail.com (A.H.); soguksup@gmail.com (P.S.); smese@istanbul.edu.tr (S.M.); aagacfidan@hotmail.com (A.A.); 2 Department of Infectious Diseases and Clinical Microbiology, Istanbul Faculty of Medicine, Istanbul University, 34093 Istanbul, Turkey; mhakankir@gmail.com (M.H.K.); acagatay@istanbul.edu.tr (A.C.); 3 Department of Medical Microbiology, Faculty of Medicine, Kırklareli University, 39100 Kırklareli, Turkey; mehmetdemirci@klu.edu.tr; 4 Department of Biostatics, Istanbul Faculty of Medicine, Istanbul University, 34093 Istanbul, Turkey; eyurt@istanbul.edu.tr (E.Y.); serraakkoyunlu@gmail.com (S.Z.A.)

**Keywords:** cytomegalovirus, HIV, opportunistic infection, CD4+ T cell, HIV-RNA

## Abstract

Cytomegalovirus (CMV) remains a major opportunistic pathogen in individuals with HIV. The aim of this study was to investigate the seroprevalence and reactivation rates of CMV among HIV-positive individuals. A total of 300 people with HIV presenting to the Istanbul Faculty of Medicine were enrolled. Serological assessments were performed using enzyme-linked immunosorbent assay (ELISA), while molecular analyses were conducted through PCR-based methods. Sociodemographic and clinical characteristics of the patients were also evaluated. Of the participants, 90% were male, with an age range of 18–76 years. Serological testing demonstrated CMV IgG positivity in 292 patients (97.3%) and CMV IgM positivity in 11 patients (4.07%). CMV DNA was detected in 91 patients (30.3%) by molecular assays, with viral loads ranging from <150 to 2,404,678 copies/mL. CMV DNA positivity was significantly more frequent in older patients (*p* < 0.05) and was associated with lower CD4+ T lymphocyte counts. CMV disease was identified in 50 patients (16.7%), with organ involvement (64%) representing the most common clinical manifestation. CMV seropositivity is remarkably high in HIV-positive individuals, and reactivation rates are increased, particularly in older patients and those with advanced immunosuppression. These findings underscore the clinical relevance of routine CMV surveillance in the management of HIV infection.

## 1. Introduction

CMV is a double-stranded DNA virus of the Herpesviridae family in the beta-herpesvirus group, exhibiting a long replication process and strict host specificity to humans [1].

Although usually asymptomatic in immunocompetent individuals, CMV reactivation can lead to clinical conditions with serious systemic and organ involvement in immunosuppressed individuals, especially people living with HIV [2]. In addition to immunity, individual differences in the development of CMV-related complications are also noteworthy. Interferon lambda 3/4 (IFNL3/4) is associated with AIDS-related CMV retinitis in individuals with HIV. Also, Interleukin-10 receptor 1 (IL-10R1) variants have been shown to affect the development of CMV retinitis in AIDS patients [3]. Before Antiretroviral Therapy (ART), CMV was one of the most common serious opportunistic infections in individuals living with HIV, but this frequency has decreased with the widespread use of ART [4].

CMV is commonly found in the community and can be transmitted through close contact with infected individuals. The main routes of transmission are saliva, urine, blood, semen, vaginal secretions, breast milk, organ transplants, and the placenta [5]. Congenital CMV infection is considered one of the leading causes of hearing loss and neurodevelopmental disorders in childhood [6]. Vertical transmission is particularly important if the mother has a primary CMV infection during pregnancy, increasing the risk of congenital CMV infection and leading to serious neurological sequelae. Transmission is also possible through the birth canal during delivery and through breastfeeding in the postpartum period [7]. Horizontal transmission is more common in infants and young children through saliva and urine, and in adults through blood and blood products, organ transplants, and unprotected sexual contact [8].

HIV targets the immune system in humans, causing immunodeficiency. The virus targets important cells of the immune system, primarily CD4+ T lymphocytes, leading to immunosuppression in infected individuals. This immunosuppression predisposes to opportunistic infections and some types of cancer. If left untreated, HIV infection progresses over time to an immunodeficiency condition called Acquired Immunodeficiency Syndrome (AIDS). There are two main types of HIV: HIV-1 and HIV-2. Although the genomes of both types are structurally similar, there are significant differences in amino acid levels. However, both types can cause AIDS [9]

HIV continues to exist as a global public healthcare concern. The first case appeared in the United States in 1981 and soon after caused a global epidemic [10].

Based on the World Health Organization (WHO) data from July 2024, approximately 39.9 million people worldwide are living with HIV. In the same year, 1.3 million new HIV cases were reported, and 630,000 people died due to HIV-related causes. Although the number of new cases has decreased as a result of the measures taken, it continues to be a serious cause of morbidity and mortality worldwide [11].

The data in Turkiye show an increase over the years. According to the Ministry of Health (MH) data from November 2024, 45,835 HIV-positive individuals and 2438 AIDS cases have been recorded in our country since the first case in 1985. Among the total HIV and AIDS cases, 81.8% were male, 18.2% were female, and 16.1% were foreign nationals. Cases were reported most frequently in the 25–29 and 30–34 age groups, respectively. Possible routes of transmission include homosexual, bisexual, and heterosexual intercourse; mother-to-child transmission; intravenous drug use; infected blood transfusion; hemophiliacs; nosocomial; multiple routes; and unknown causes. Transmission due to unknown causes is the most common route of transmission, with a rate of 58.9% [12].

In this context, the present study aimed to determine the seroprevalence and reactivation rates of CMV in individuals living with HIV who applied to our hospital, and to examine the relationship between the presence of detected CMV and the demographic characteristics, immunological parameters, and clinical results of the patients.

## 2. Materials and Methods

The study had a retrospective cross-sectional design and was conducted with the data from 300 people living with HIV who presented to the Infectious Diseases and Clinical Microbiology Clinic of Istanbul University, Istanbul Faculty of Medicine Hospital between 2020 and 2024. Individuals over 18 years of age were included in the study. Among these patients, 270 (90%) were male, and 30 (10%) were female.

The study included individuals who were confirmed to be HIV positive using serological and molecular methods. Their CMV serological profiles and CMV DNA results were evaluated for co-infection. Additionally, CD4+ T cell counts and HIV RNA levels were examined, and clinical results related to CMV were recorded. CMV IgG, CMV IgM and CMV DNA-PCR data were used for CMV infection diagnosis. For CMV disease, clinical/imaging/pathology findings were used in addition to CMV DNA positivity. To ensure clinical correlation, all diagnoses of CMV end-organ disease were clinically and radiologically validated within 30 days following the laboratory detection of CMV DNAemia.

CMV IgG and IgM serological tests were performed using a Triturus^®^ fully automated ELISA device (Grifols), and Vircell^®^ CMV IgG and IgM ELISA kits (Vircell, Spain) were used in tests. The working principle of the CMV IgG test is based on the reaction of antibodies in the tested serum with polystyrene surfaces coated with the antigen of the ATCC VR-538 strain. The working principle of the CMV IgM ELISA test is based on the capture of IgM in the sample by anti-IgM antibodies adsorbed on the polystyrene surface. Immunoglobulins that do not bind to the surface are removed from the medium in the washing stage. In the second stage, anti-human globulin binds to the antigen–antibody complex, and after the addition of substrate and incubation time, a color change is observed.

Quantitative CMV DNA test results of the patients were obtained from the data in the patient files and evaluated. A fully automated Cobas^®^ 6800 system (Roche) was used in the study. According to the device’s operating principle, plasma samples from patients are placed in the sample loading unit. Samples are automatically identified by their barcodes. Viral DNA is isolated from the sample by automatic nucleic acid extraction performed by the device. The isolated nucleic acid is mixed with the PCR master mix within the system. Primers, enzymes, and probes are added to the reaction mixture. The target nucleic acid is amplified using the Real-Time PCR method. The amount of amplified DNA is measured in each cycle using fluorescent signals, and Ct (cycle threshold) values are determined at this stage. The system analyzes the amplification curves and shows positive, negative, or suspicious results. In this system used for molecular analysis, a lower limit of quantification (LOQ) of approximately 34.5 IU/mL has been established for CMV DNA. The entire process is automatically evaluated within the system.

Statistical analyses were made with the SPSS software (version 30.0, IBM Corp.). The normality of the data distributions was examined using the Kolmogorov–Smirnov or Shapiro–Wilk test, depending on the sample size. Since no normal distribution was detected, non-parametric tests were applied. The *p*-values for age, CD4, CD8, and CD4/CD8 variables were obtained using the Mann–Whitney U test. The *p*-values for other variables were obtained using the Chi-Square test.

The study was conducted in line with the principles of the Helsinki Declaration and was approved by the Academic Board of the Department of Medical Microbiology, Istanbul Faculty of Medicine, Istanbul University, on 28 April 2025, with the ethics committee decision (Prot. N. 3293212).

## 3. Results

The study included 300 people with HIV who presented to the Istanbul Faculty of Medicine Hospital. In total, 270 of the patients were male (90%) and 30 were female (10%), with an age range of 18–76 years. Blood samples were analyzed serologically using the ELISA method. Serological test results showed that 292 patients (97.3%) were positive for CMV IgG and 11 patients (4.07%) were positive for CMV IgM. These results indicate a low rate of acute infection.

As shown in Table 1, analysis of the molecular test data revealed CMV DNA positivity in 91 (30.3) patients. The CMV DNA viral load values of these patients ranged from <150 to 2,404,678 copies/mL. These results were interpreted as reactivation of latent CMV infection in individuals living with HIV.

A total of 265 out of 270 male patients (98.15%) tested positive for CMV IgG, while CMV IgM positivity was observed in 11 patients (4.07%). In women, CMV IgG positivity was detected in 27 out of 30 patients (90%), while CMV IgM positivity was not determined. CMV DNA positivity was present in 85 male patients (31.5%), while it was recorded in 6 female patients (20%). We requested an avidity test in cases where CMV infection was suspected, and both IgM and IgG were positive. In the two cases we defined as acute CMV, our anti-CMV IgG avidity was found to be low (Table 1).

The 300 patients included in the study were divided into 3 age groups as follows.

(1) 18–30 (*n* = 72)
(2) 31–50 (*n* = 154)
(3) 51–76 (*n* = 74)

In the 18–30 age group, CMV IgG positivity was detected in 69 (95.83%), CMV IgM positivity in 2 (2.78%), and CMV DNA positivity in 18 (25%) individuals. In the 31–50 age group, CMV IgG was found in 152 (98.7%) individuals, CMV IgM positivity in 7 (4.55%), and CMV DNA positivity in 43 (27.92%) individuals. In the 51–76 age group, CMV IgG positivity was detected in 71 (95.95%) individuals, CMV IgM positivity in 2 (2.7%), and CMV DNA positivity in 30 (40.54%) individuals.

Although no significant differences were detected in CMV IgG and IgM positivity rates among age groups (*p* > 0.05), CMV DNA positivity was higher in the older age group (51–76 years) compared to other groups. This suggests that the risk of reactivation of latent CMV infection may increase with age (Table 1).

The sociodemographic characteristics of the study population are summarized in Table 2. The majority of participants were residing in Istanbul (86%), while 14% lived in other provinces. Regarding sexual orientation, most individuals identified as heterosexual (58.66%), followed by men who have sex with men (MSM) (26.67%) and bisexual individuals (11%). A small proportion reported transsexual identity (1%), no sexual activity (0.67%), or unknown orientation (2%). In terms of educational status, primary education (35.33%) and postgraduate education (30.33%) constituted the largest groups. More than half of the patients were single (51.3%), whereas 45% were married and 3.7% were divorced.

Various opportunistic infections and AIDS-defining illnesses were observed in 50 HIV-positive patients with CMV DNA positivity. The most common opportunistic infection was Pneumocystis jirovecii pneumonia (PCP), detected in eight patients. Tuberculosis was detected in five patients, and disseminated tuberculosis in one patient. Candida esophagitis was observed in seven patients, and oral candidiasis in one patient. Kaposi’s sarcoma was detected in three patients, progressive multifocal leukoencephalopathy (PML) in four patients, and toxoplasma encephalitis in three patients. Additionally, cryptococcal infection was detected in one patient, lymphoma in one patient, central nervous system lymphoma in one patient, Salmonella infection in one patient, Castleman’s disease in one patient, and MAC infection along with herpes zoster in one patient.

These findings indicate advanced immunosuppression and a high burden of opportunistic infections in CMV-positive HIV patients.

### 3.1. Variables Showing Significant Differences with CMV DNA

Statistically significant differences were detected after comparing CMV DNA-positive and negative individuals. The mean age of CMV DNA-positive patients was 44.87, while that of negative individuals was 39.94 (*p* = 0.003, effect size = 0.213). CD4+ T cell counts were significantly lower in some of the individuals living with HIV (*p* < 0.0001, effect size = 0.685). CD8+ T cell levels were similarly low (*p* < 0.0001, effect size = 0.359). A significant decrease was detected in the CD4/CD8 ratio (*p* = 0.000, effect size = 0.0646). Differences were also found in positivity rates in terms of sexual orientation (*p* = 0.037, effect size = 0.204), with the highest rate among those whose sexual orientation was unknown (83.3%), followed by bisexual (36.4%) and heterosexual (31.3%) individuals, respectively.

### 3.2. Variables with Significant Differences with CMV IgM

A significant relationship was detected between CMV IgM and CMV DNA positivity in the province of residence (*p* = 0.019, effect size = 0.419). These rates were particularly observed in Tokat, Izmir, Kocaeli, Hatay, and Sakarya. A significant difference was observed between sexual orientation (*p* = 0.003, effect size = 0.284). The positivity rate was 36.4% in bisexual individuals, 6.2% in men who have sex with men (MSM), and 5.3% in heterosexual individuals. CMV IgM positivity was not found in transsex, non-sexual, and groups with unknown sexual orientation. No significant differences were found in age, sex, education level, and marital status variables.

### 3.3. Comparison of Variables According to CMV IgG Seropositivity

There were no significant differences in CMV IgG positivity among the variables in our analyses. This indicates that the infection experienced by the individuals included in the study occurred independently of demographic factors such as age, sex, and sexual orientation.

The mean age of the 50 patients who were included in the study was 46.8 ± 11.9 years, and the vast majority of patients were male (92%). The median CD4 cell count, reflecting the immune status of the cases, was found to be 38 cells/mm^3^ (1–759), indicating that the study group was under significant immunosuppression. The very high median HIV RNA level (597,322 copies/mL) revealed active viral replication, while the median CMV DNA value was 5598 copies/mL, showing a wide distribution. These results suggest that severe immunodeficiency and high viral load constitute a significant risk for CMV DNAemia in the study group, as shown in Table 3.

It was observed in the clinical diagnosis distribution of the patients that the most common condition was pneumonia (30%), followed by viremia without organ involvement (26%) and CMV syndrome (16%). Among pneumonia patients, seven cases were classified as probable CMV pneumonia and eight cases as highly probable CMV pneumonia according to predefined diagnostic criteria. In these patients, the HIV viral load was found to be in the range of 18,545–13,162,479 copies/mL. Retinitis (10%) and colitis (8%) were detected less frequently, while serious organ involvements such as encephalitis (4%), esophagitis (4%), and myelitis (2%) were observed less frequently. These results indicate that CMV infection in the study group follows a wide clinical spectrum, with respiratory system and systemic involvement being particularly prominent, as shown in Table 4.

It was found in the treatment protocols of the patients that the most frequently applied approach was sequential treatment with ganciclovir followed by valganciclovir (54%). Among the cohort, 22% of the patients were monitored without treatment, and death occurred during ganciclovir treatment in 12% of cases. Cases treated with oral valganciclovir alone accounted for 6%, while foscarnet and ganciclovir combined with plasmapheresis treatments with foscarnet and ganciclovir were applied less frequently (2% each). In addition, 2% of patients died before diagnosis. This distribution indicates that intravenous antiviral therapy constituted the primary treatment approach in the study and that mortality was clinically significant in the patient group (Table 5). In accordance with our clinical protocol for patients with CMV organ involvement, ART initiation was generally deferred until the completion of the anti-CMV induction phase to mitigate the risk of Immune Reconstitution Inflammatory Syndrome (IRIS).

This study comprehensively investigated the relationships between patients’ clinical results, underlying disease parameters, and mortality. The association of clinical results (fever, diarrhea, lymphadenopathy, etc.) with both CD4 cell count and HIV RNA viral load, which are key indicators of HIV infection, and mortality was analyzed using non-parametric statistical methods. The analyses revealed that only lymphadenopathy was significantly associated with CMV IgM seropositivity (Fisher’s Exact Test, *p* = 0.046, effect size = 0.515). No statistically significant association was observed between other clinical results and CD4 levels, HIV RNA levels, or mortality, nor between mortality and the examined immunological parameters or complications. This result suggests that lymphadenopathy may be a possible clinical indicator of CMV reactivation or infection in our study population, while the inability to demonstrate other expected associations can be explained by factors such as the homogeneous clinical course of our patients or the sample size.

CMV disease was detected in 50 (16.66%) of 300 patients in the present study, with an average age of 41.39 years. Among these, 46 (92%) were male, and 4 (8%) were female. The average CD4+ T lymphocyte count in those who had CMV disease was 160.31, which was lower than expected. Except for one individual, the clinical presentations in all individuals with CMV disease were associated with CMV reactivation; only two patients showed acute CMV infection. The average CD4+ T lymphocyte count in these patients was 744 cells/mm^3^, and they did not receive treatment. When clinical presentations were evaluated across the 50 patients, organ involvement was observed in 32 patients, CMV syndrome in 6, and CMV viremia in 12. Patients with CMV viremia did not show clinical symptoms, and for these cases, treatment was determined individually by the clinician, as there is no clear universal recommendation for asymptomatic viremia.

The immunological profile of CMV DNA-positive patients showed severe immunosuppression. In 90% of patients, the CD4 count was below 200 cells/µL, and in more than half, the CD4 level was below 50 cells/µL. Additionally, 90% of patients had a CD4/CD8 ratio below 0.3, indicating severe immune dysregulation. These findings support a strong association between advanced immunosuppression and CMV reactivation in individuals with HIV (Table 6).

## 4. Discussion

As one of the seven herpesviruses of the Herpesviridae family, CMV is an important opportunistic infection that can remain latent after infection and has reactivating properties. Although morphologically similar to other members of the herpesviridae family, it can be distinguished by its antigenic differences [10]. CMV is common in the population, and transmission occurs through close contact. The main transmission occurs through saliva, urine, blood, semen, and placenta [5]. Globally, the seropositivity rate is between 40 and 100%, but may vary regionally. While the infection is mostly asymptomatic in healthy individuals, it can cause serious complications such as systemic and organ involvement in immunosuppressed individuals [13]. The main organ involvement sites are the eyes, digestive system, lungs, liver, and central nervous system [14]. In the presence of advanced HIV infection, CMV becomes clinically significant with a significant decrease in CD4+ T cell count [15]. The high seropositivity rate of 97.3% found in our study for CMV IgG indicates that CMV infection is common among individuals living with HIV and is consistent with global seroprevalence rates.

CMV viremia was found to increase with age in the study (*p* = 0.028, effect size = 0.154), which is consistent with previous studies on the subject [16]. As seen in our study, with the emergence of current HAART regimens, the increase in treatment rates, the availability of early diagnosis, and the decrease in the number of patients in the AIDS stage, CMV reactivation and consequently CMV viremia are seen less frequently [17]. In our study, the higher rate of CMV DNA positivity, especially in the 51–76 age group (40.54%), supports the idea that the risk of reactivation of latent CMV infection increases with age.

CMV pneumonia is the most common type of organ involvement in individuals with CMV disease [18]. Other involvements are identified as CMV retinitis, CMV colitis, CMV encephalitis, and CMV myelitis, respectively. CMV syndrome is diagnosed by weakness, fever, atypical lymphocytosis, leukopenia or neutropenia, thrombocytopenia, and elevated liver function tests (LFTs), along with the detection of CMV DNA in the blood [19]. CMV pneumonia is diagnosed by the presence of pneumonia clinical results, in addition to thoracic CT imaging consistent with the clinical picture, CMV viremia, and positive CMV DNA in BAL samples [20]. In our study, organ involvement was observed in a significant proportion of cases diagnosed with CMV disease, and the most frequent clinical presentations were organ involvement and CMV syndrome, which is consistent with the literature data. While ‘CMV syndrome’ is a terminology more frequently utilized in solid organ transplant settings, it is increasingly recognized in the literature as a distinct clinical entity for people with AIDS who present with systemic symptoms like fever and cytopenia in the absence of localized organ disease.

Previous studies reported that CMV colitis is observed in addition to gastroenteritis symptoms [21]. In our study, CMV colitis was observed in four patients, and the diagnosis was determined by detecting a pathological appearance consistent with CMV infection in the colon biopsy. This result reveals that CMV colitis is an important clinical condition in individuals living with HIV and is consistent with the data reported in the literature. CMV encephalitis and myelitis diagnoses are made in addition to the patient’s clinical condition, with CSF results consistent with viral encephalitis (lymphocytic pleocytosis, increased protein, hypoglycohorchia), CMV DNA positivity, and compatibility of imaging results [22]. Similar results were obtained in our study. This shows that the diagnostic approach in our study is consistent with the criteria recommended in the literature. CMV retinitis is diagnosed by detecting viremia in the blood and eye examination [14]. The same diagnostic algorithm was applied to patients with CMV retinitis in our study. Ganciclovir and valganciclovir were classically used in the treatment of patients. In our study, the application of standard treatment protocols in patients diagnosed with CMV retinitis indicates that clinical management was consistent with current guidelines.

A study conducted in Nigeria by Fowotade et al. (2015) investigated the prevalence of CMV infection in HIV/AIDS-positive patients. This study, conducted with 180 patients (108 women, 72 men) aged 16–56 years, found that 93.9% of patients were positive for CMV IgG and 11.1% were positive for CMV IgM, indicating active infection. CMV seropositivity was associated with age, marital status, number of sexual partners, CD4+ T lymphocyte count, and previous blood transfusions. No significant association was found with occupation, sex, or ART. In conclusion, it was shown that CMV infection is common in patients living with HIV and that CMV is hyperendemic in HIV-positive individuals in this region [23]. In our study, the CMV IgG positivity rate was found to be 97.3%, and the CMV IgM positivity rate was 4.07%, indicating a low rate of acute infection and a widespread latent infection, which is largely consistent with this study.

A retrospective study was conducted by Borcak et al. between 2016 and 2022 in a hospital in Turkiye with 36 individuals who were positive for CMV DNA among 115 HIV/AIDS patients (31,3%). In the study, CD4+ T lymphocyte counts, CMV DNA viral load values, and CMV IgM and IgG antibody levels were recorded to determine risk factors for CMV. CMV disease was detected in 18 patients, while CMV infection was observed in the other 18 patients. Of these patients, 31 were male, and their average age was 46 ± 13. In the group with CMV disease, the CD4+ T lymphocyte count was low, while the HIV viral load was high. In serological evaluation, the rate of CMV IgM positivity was found to be 5.5%, and it was observed that CMV IgM positivity was lower in the diseased group compared to the infected group. All individuals in both groups were determined to be CMV IgG positive. The CMV viral load threshold value was found to be 3154 copies/mL [24]. Our study found significantly lower CD4+ T lymphocyte counts and CD4/CD8 ratios in CMV DNA-positive individuals, supporting the results of Borcak et al.

A retrospective study that was conducted by Chao et al. between 2017 and 2019 in 808 HIV/AIDS patients in China detected CMV infection. HIV RNA, CD4+ T lymphocyte counts, CMV DNA, and antibodies were investigated in the patients. CMV DNA positivity rates were compared in blood, urine, and CSF samples. The correlation between immune status and CMV positivity was analyzed using CD4+ T lymphocyte count. According to the study results, CMV infection positivity was found to be 29.05%. CMV DNA positivity rates in blood, urine, and CSF samples were found to be 25.27%, 26.01%, and 5.70%, respectively, with no significant difference between blood and urine. However, a significant difference was determined between CMV IgM antibody levels and CMV DNA results in urine and blood samples. The researchers stated that low CD4+ T lymphocyte counts and high HIV viral loads are risk factors for CMV infection among HIV/AIDS patients [25]. In our study, CMV DNA positivity was detected at 30.3%, which is consistent with the results reported by Chao et al.

In another study conducted in Africa in 2017 by Grønborg et al., CMV seroprevalence was reported to be higher than 90% in individuals with HIV. The most common symptoms were identified as retinitis, pneumonia, and colitis, and these clinical presentations were associated with low CD4+ counts. They also found that the prognosis and increased risk of transmission of HIV infection in these patients were associated with CMV. In addition, the detection of CMV DNA in samples was shown to be an independent risk factor for HIV transmission and mortality [26]. The result of CMV IgG seropositivity at 97.3% in our study is consistent with this study. In our cohort, pneumonia emerged as the most frequent manifestation of CMV end-organ disease. While regional variations exist, similar clinical involvements have been reported in other Eastern European cohorts, albeit at lower frequencies; for instance, Halichidis et al. reported a case of pneumonia within their study group [18]

Opportunistic infections caused by HIV were evaluated in a case report from Turkiye in 2024 by Şahin et al. The study was conducted with a 48-year-old female and a 40-year-old male patient, and HIV RNA viral loads were recorded as 94,500 copies/mL and 755,200 copies/mL, respectively. The presence of CMV was confirmed in both patients as a result of laboratory examination. In the female patient, the CD4+ T lymphocyte count was 30 cells/mm^3^, CMV IgM positive and CMV IgG positive, while in the male patient, the CD4+ T lymphocyte count was 7 cells/mm^3^, CMV IgM negative and CMV IgG positive. CD4/CD8 ratios were recorded as 0.02 and 0.05, respectively. It was emphasized that high HIV viral loads and low CD4+ counts pose a risk for HIV/AIDS patients, and it was recommended that the CMV agent be considered in immunosuppressed individuals [27]. In our study, the significantly lower CD4+ T lymphocyte counts in individuals who developed CMV disease are consistent with the risk factors highlighted in these case reports.

Halichidis et al. (2023) aimed to determine the prevalence of CMV infection among women with HIV in southeastern Romania. The study included 50 out of 70 women with HIV (mean age 33). After administering a questionnaire regarding their social conditions and related illnesses, they investigated the correlation between HIV status, CD4+ T lymphocyte counts, viral load, and ART (Antiretroviral Therapy). It was found that 29 cases were diagnosed with HIV after the onset of sexual activity, and 21 cases were diagnosed before sexual activity. Most patients had CD4+ T lymphocyte counts above 200 cells/µL, and the duration of ART was shorter in these patients compared to those with CD4+ T lymphocyte counts <200 cells/µL. A total of 41 patients showed no viremia, and their mean CD4+ T lymphocyte count was 704.71 cells/µL. In patients with detected viremia, this number was found to be 452.44 cells/µL. A positive correlation was found between CMV IgG values and ART duration. A negative correlation was found between CMV IgG and CD4+ T lymphocyte count. In conclusion, it was determined that CMV IgG values increased as ART duration increased, while both CMV IgG levels and CMV avidity increased as CD4+ T lymphocyte count decreased [18]. In our study, CMV IgG positivity was independent of demographic variables, and the relationship between CMV DNA positivity and immunosuppression is consistent with this study.

Another study was conducted by Albasanz-Puig et al. between 2015 and 2018 to evaluate CMV replication and CMV-specific immune response in patients with advanced HIV infection after initiation of ART. This study included 53 patients with CD4+ T lymphocyte counts <100 cells/µL. All patients received ART. At baseline, the median CD4+ T lymphocyte count was 30 cells/µL, and the HIV viral load was 462,000 copies/mL. CMV viremia was detected in 32% of the patients. During the follow-up period, only one patient experienced CMV-related organ disease. Seven patients died from non-CMV-related causes during the study period. The CMV-specific immune response was initially found to be 71.7%, and this rate remained at 70.0% at week 48. As a result of the study, although CMV viremia is initially common in individuals with advanced HIV infection, CMV replication was suppressed with immunological improvement after ART, and a decrease in the risk of developing organ disease was recorded [28]. The absence of clinical progression in most of the patients with asymptomatic CMV viremia in our study supports the suppressive effect of ART on CMV replication.

Although the single-center and retrospective nature of the present study caused some limitations, the large sample size of 300 individuals included and the detailed analysis of the data using both serological and molecular methods make our study strong and unique in reflecting the epidemiology of CMV in the HIV-positive population in our country.

## 5. Limitations

A notable limitation of the present study is the absence of comprehensive and routine Epstein–Barr Virus (EBV) DNA PCR testing data across the patient cohort. In standard clinical practice, routine monitoring of EBV viral load is not recommended by established CMV diagnosis and management guidelines. This is primarily because concurrent EBV status is not currently recognized as a determining factor for clinical diagnosis, overall prognosis, or the immediate therapeutic management of these patients. Consequently, clinicians do not routinely request EBV PCR panels, as the results rarely alter established treatment protocols. While this lack of data accurately reflects real-world clinical workflows, it inherently limits our study’s capacity to perform a more in-depth evaluation of potential CMV/EBV co-reactivation dynamics and their synergistic effects on patient outcomes.

## 6. Conclusions

The results of the present study confirm the prevalence of latent infection in individuals living with HIV, with a high seroprevalence rate of 97.3%, and reveal a significant increase in CMV DNA positivity, particularly in older patients and those with low CD4+ T lymphocyte levels. The increased risk of reactivation as immunosuppression deepens highlights the vital importance of regular virological monitoring in this patient group.

In this context, our study aimed to contribute to the national literature by strengthening the epidemiological data on CMV infection in individuals living with HIV in our country. Given that CMV can lead to serious mortality and morbidity in immunosuppressed individuals, it is hoped that our results will guide the development of early diagnosis strategies, the optimization of treatment protocols, and clinical practices in at-risk groups.

## Figures and Tables

**Table 1:** Distribution of CMV IgG, CMV IgM, and CMV DNA positivity by sex and age groups.

Characteristics	Number of Patients (*n*)	Percentage (%)			
**Total Patients**	300	100			
**Sex**			**CMV IgG (+)**	**CMV IgM (+)**	**CMV DNA (+)**
Male	270	90	265 (98.15%)	11 (4.07%)	85 (31.5%)
Female	30	10	27 (90%)	0	6 (20%)
**Age Groups**					
18–30	72	24	69 (95.83%)	2 (2.78%)	18 (25%)
31–50	154	51.33	152 (98.7%)	7 (4.55%)	43 (27.92%)
51–76	74	24.67	71 (95.95%)	2 (2.7%)	30 (40.54%)

**Table 2:** Sociodemographic characteristics and CMV DNA distribution among the study group (*n* = 300).

Variables	Total *n* (%)	CMV DNA (+)	CMV DNA (-)	Positivity Rate *
**Residence**				
Istanbul	258 (86.0)	78	180	30.2%
Other Provinces	42 (14.0)	13	29	31.0%
**Sexual Orientation**				
Heterosexual	176 (58.7)	55	121	31.2%
MSM	80 (26.7)	19	61	23.7%
Bisexual	33 (11.0)	12	21	36.4%
Unknown	6 (2.0)	5	1	83.3%
No Sex	2 (0.7)	0	2	0.0%
Transsexual	3 (1.0)	0	3	0.0%
**Education Level**				
Primary education	106 (35.3)	34	72	32.1%
Middle school	13 (4.3)	3	10	23.1%
High school	89 (29.7)	32	57	36.0%
Higher education **	92 (30.6)	22	70	23.9%
**Civil Status**				
Married	135 (45.0)	41	94	30.4%
Single	154 (51.3)	46	108	29.9%
Divorced	11 (3.7)	4	7	36.4%

* Internal group positivity rate. ** Combined undergraduate and post-graduate data.

**Table 3:** Demographic and clinical characteristics of patients with CMV infection.

Variables	Value
Total Number of Patients	50
Age (Mean ± SD)	46.8 ± 11.9
Age (Median [Min–Max])	48 [26–74]
Sex: Male *n* (%)	46 (92.0%)
Sex: Female *n* (%)	4 (8.0%)
CD4 Count (cells/mm^3^) (Median [Min–Max])	38 [1–759]
HIV RNA (copies/mL) (Median [Min–Max])	597,322 [143–53,200,000]
CMV DNA (copies/mL) (Median [Min–Max])	5598 [55–2,404,578]

**Table 4:** Distribution of CMV organ involvement.

Diagnoses	Numbers	Percentages (%)
Pneumonia	15	30.0%
Viremia (without organ involvement)	13	26.0%
CMV Syndrome (Acute CMV Syndrome)	8	16.0%
Retinitis	5	10.0%
Colitis	4	8.0%
Encephalitis	2	4.0%
Esophagitis	2	4.0%
Myelitis	1	2.0%

**Table 5:** Treatment approaches and outcomes.

Treatment Protocol	Numbers	Percentages (%)
Ganciclovir → Valganciclovir (Sequen-	27	54.0%
tial Treatment)		
No Treatment Received/Follow-up	11	22.0%
Ganciclovir (Death during treatment)	6	12.0%
Valganciclovir (Oral treatment only)	3	6.0%
Foscarnet	1	2.0%
Ganciclovir + Plasmapheresis	1	2.0%
Death before diagnosis	1	2.0%

**Table 6:** Immunological parameters (CD4, CD8, and CD4/CD8 ratio) of the CMV DNA-positive patient group (*n* = 50).

Parameters	*n*	%
CD4 Count (cells/µL)		
<50 cells/µL	28	56.0
51–199 cells/µL	17	34.0
200–499 cells/µL	3	6.0
>500 cells/µL	2	4.0
CD8 Count (cells/µL)		
0–399 cells/µL	16	32.0
400–899 cells/µL	22	44.0
900–1499 cells/µL	11	22.0
>1500 cells/µL	1	2.0
CD4/CD8 Ratio		
<0.3	45	90.0
0.31–0.9	4	8.0
0.91–1.0	0	0.0
>1.0	1	2.0

## Data Availability

The data presented in this study are available upon request from the corresponding author.
